# The endocrine disruptor chlorpyrifos alters hypothalamic Npy and Agrp expression via ERβ-dependent regulation *in vitro* and *in vivo*

**DOI:** 10.3389/fendo.2025.1726498

**Published:** 2026-01-14

**Authors:** Monica Pastorino, Antonella Desiderio, Erica Perrella, Michele Campitelli, Cecilia Nigro, Teresa Peluso, Mario De Felice, Concetta Ambrosino, Francesco Beguinot, Claudia Miele, Gregory Alexander Raciti

**Affiliations:** 1 Department of Translational Medicine, Federico II University of Naples, Naples, Italy; 2 Institute of Endotypes in Oncology, Metabolism and Immunology, National Research Council, Naples, Italy; 3 Biogem Scarl, Institute of Molecular Biology and Genetics Research, Ariano Irpino, Italy; 4 Department of Molecular Medicine and Medical Biotechnology, Federico II University of Naples, Naples, Italy; 5 Department of Biology, Federico II University of Naples, Naples, Italy

**Keywords:** agouti-related peptide, chlorpyrifos, endocrine disruptors, estrogen receptor beta, hypothalamus, metabolic disruption, Neuropeptide Y, orexigenic neuropeptides

## Abstract

**Introduction:**

Obesity represents a global health concern, with the hypothalamus playing a central role in regulating energy balance. Chlorpyrifos (CPF), a widely used organophosphate pesticide, is now recognized as an endocrine-disrupting chemical (EDC). Although the peripheral metabolic effects of CPF are relatively well characterized, its potential impact on central energy balance remains to be investigated. Here, we examined, both *in vitro* in murine hypothalamic cells and *in vivo* in murine hypothalami, whether CPF modulates the orexigenic mediators Neuropeptide Y (Npy) and Agouti-related peptide (Agrp). We further explored the molecular mechanisms underlying this regulation.

**Methods:**

For *in vitro* studies, murine mHypoE-N46 hypothalamic cells were treated with CPF (1 pM) under acute (4 h) and chronic (6-day) exposure conditions. For *in vivo* studies, hypothalamic tissue from CD-1 mice chronically exposed to CPF (10 mg/kg/day), from conception to 6 months of age while maintained on a standard diet, were analyzed. Gene and protein expression levels, as well as neuropeptide secretion, were assessed by qPCR, Western blotting, and ELISA, respectively. Selective pharmacological antagonists of ERβ and ERα were employed to determine receptor-specific effects.

**Results:**

Both acute and chronic CPF exposure significantly increased *Npy* and *Agrp* mRNA expression and secretion in mHypoE-N46 cells. Chronic CPF treatment selectively upregulated *ERβ* at the mRNA and protein levels, whereas *ERα* was unaffected. Pharmacological inhibition confirmed ERβ as the main mediator of CPF action. Consistently, *in vivo* hypothalamic samples from CPF-exposed mice displayed increased *Npy* and *Agrp* expression and an elevated *ERβ/ERα* ratio, mirroring the *in vitro* findings. Also, CPF exposure increased *leptin receptor* (*Lepr*) expression both *in vitro* and *in vivo*.

**Conclusion:**

Our findings indicate that CPF acts as a central endocrine disruptor by shifting estrogen receptor signaling toward ERβ dominance, thereby promoting orexigenic neuropeptide expression. Together with the increased *Lepr* expression, this mechanism may contribute to hypothalamic dysfunction and metabolic imbalance. The evolutionary conservation of hypothalamic circuits suggests that similar mechanisms may operate across species and impact metabolic health.

## 1 Introduction

Obesity is a complex, multifactorial disease with a rapidly increasing prevalence worldwide, imposing a significant burden on healthcare systems (1, 2). It results from an imbalance between energy intake and expenditure and is driven by environmental determinants, including the obesogenic food environment, sedentary lifestyles, and exposure to environmental chemicals, while genetic background shapes individual susceptibility (3–5). Within this environmental context, growing attention has focused on endocrine-disrupting chemicals (EDCs), which interfere with hormonal signaling, thereby affecting energy balance and promoting obesity (5–10). Among EDCs, bisphenol A (BPA), widely used in plastics and resins and commonly detected in food containers and other consumer products, is the most extensively studied (11–13). Evidence from both epidemiological and experimental studies has consistently linked BPA exposure to obesity, leading to progressive regulatory restrictions in several applications, particularly within the European Union (14). For instance, the National Health and Nutrition Examination Survey-based analyses consistently report positive associations between urinary BPA levels and obesity in both adults and adolescents, while developmental exposure in animal models increases adiposity and obesity risk in offspring (15–17).

Organophosphate pesticides, a class of neurotoxic compounds, have also been shown to possess endocrine-disrupting properties and are emerging as contributors to metabolic diseases (18). Among them, chlorpyrifos (CPF) is one of the most widely used and is primarily known for its neurotoxic effects through acetylcholinesterase inhibition (19, 20). CPF is a highly lipophilic compound (log P ≈ 4.7) with a relatively small molecular weight (350.6 g/mol), properties that facilitate membrane partitioning, passive diffusion across biological barriers, intracellular uptake without carrier-mediated transport, and bioaccumulation in lipid-rich tissues (21). Human exposure occurs predominantly via dietary intake, although indoor air inhalation and occupational dermal contact may also contribute (20, 22). Environmental biomonitoring studies have detected CPF in personal air samples, maternal blood, and umbilical cord blood during pregnancy, demonstrating continuous low-dose exposure even in non-occupational settings (23, 24). Following absorption, CPF is rapidly metabolized, primarily in the liver, to chlorpyrifos-oxon, its bioactive metabolite, and to 3,5,6-trichloro-2-pyridinol (TCPy), which is commonly measured in urine as a biomarker of exposure (20, 22). CPF may also undergo hydrolysis to diethylphosphate (DEP), diethylthiophosphate (DETP), and other dialkyl phosphate or dialkylthiophosphate metabolites, which can be conjugated and excreted mainly through urine (20, 22). CPF metabolism is tissue- and matrix-dependent, varying according to whether the compound is present in biological tissues, food, water, or plant matrices, and human studies indicate a rapid clearance, with a reported half-life of approximately 24–27 hours (20, 25). Toxicogenomic evidence demonstrates that even low-dose CPF exposure activates xenobiotic metabolism, oxidative stress, and inflammatory pathways, leading to measurable phenotypic outcomes *in vivo* (26). Beyond peripheral metabolism, CPF’s marked lipophilicity allows it to cross the blood-brain barrier directly, a process supported by *in vitro* barrier models such as endothelial-astrocyte co-cultures and RBE4 monolayers that have shown CPF passage and intracellular detection (27, 28). Although the contribution of circulating metabolites cannot be excluded, current evidence suggests that CPF can enter the central nervous system, with documented neurotoxicological implications (19, 20).

Beyond its canonical neurotoxic effects, evidence indicates that CPF can also interfere with endocrine pathways. For instance, in CD-1 mice, developmental low-dose exposure to CPF alters thyroid function and thyroid hormone levels (29). We further showed that developmental and lifelong exposure to CPF disrupts thyroid hormone signaling in the liver, leading to sex- and generation-specific alterations in glucose homeostasis via activation of the triiodothyronine-forkhead box protein O1 axis (30). Other studies in mice have likewise demonstrated that low-dose CPF exposure generally promotes obesity and metabolic dysfunction (31, 32). In adulthood, CPF exposure leads to an obese-diabetic-like profile in apoE3 mice, characterized by excessive weight gain associated with increased food intake as well as elevated glucose, insulin, and total cholesterol concentrations (31). Similarly, dietary exposure to CPF promotes weight gain in mice fed a high-fat diet under thermoneutral conditions by impairing mitochondrial function and reducing thermogenesis in brown adipose tissue (32). Altogether, these findings establish CPF as a metabolic disruptor in addition to its well-recognized neurotoxicity. In light of mounting evidence of its adverse effects on human health and the environment, the European Union has recently adopted a ban on CPF (33, 34).

The hypothalamus integrates peripheral and central cues to regulate energy balance, with the arcuate nucleus (ARC) acting as a key hub. Within the ARC, orexigenic NPY/AgRP neurons stimulate appetite and energy storage, whereas anorexigenic pro-opiomelanocortin (POMC) neurons/cocaine- and amphetamine-regulated transcript (CART) neurons exert opposing effects by promoting satiety and energy expenditure (35). NPY and AgRP are among the most potent drivers of feeding behavior. NPY rapidly increases during fasting to enhance food intake, while AgRP provides sustained orexigenic drive by antagonizing melanocortin receptors (35–38). Through these mechanisms, NPY/AgRP neurons not only stimulate food intake but also suppress anorexigenic pathways, amplifying their action (39). Experimental studies have shown that loss of NPY/AgRP neurons reduces feeding, whereas their activation elicits marked hyperphagia, underscoring their central role in energy homeostasis (40–42).

In this study, we show that CPF directly targets hypothalamic orexigenic signaling. Using both *in vitro* and *in vivo* models, we demonstrate that CPF increases *Npy* and *Agrp* expression (*in vitro* and *in vivo*) and secretion (*in vitro*), at least in part through ERβ-dependent mechanisms. These findings provide new mechanistic insight into the impact of CPF on hypothalamic orexigenic regulation and strengthen its role as a metabolic disruptor.

## 2 Materials and methods

### 2.1 mHypoE-N46 cell culture, treatments, and SRB assay

mHypoE-N46 cells were obtained from the American Type Culture Collection (ATCC, Manassas, VA, USA; RRID: CVCL_D459) and cultured in Dulbecco’s Modified Eagle’s Medium (DMEM, 4.5 g/L glucose; Sigma-Aldrich, St. Louis, MO, USA), supplemented with 10% fetal bovine serum (FBS; Thermo Fisher Scientific, Waltham, MA, USA), 100 U/mL penicillin, and 100 µg/mL streptomycin (Lonza, Walkersville, MD, USA). Cells were maintained at 37°C in a humidified atmosphere containing 5% CO_2_. Chlorpyrifos (CPF), 4-[2-Phenyl-5,7-bis(trifluoromethyl)pyrazolo[1,5-a]pyrimidin-3-yl]phenol (PHTPP), and 1,3-Bis(4-hydroxyphenyl)-4-methyl-5-[4-(2-piperidinylethoxy)phenol]-1H-pyrazole-dihydrochloride (MPP) were purchased from Sigma-Aldrich, and stock solutions were prepared in dimethyl sulfoxide (DMSO; Sigma-Aldrich). For CPF treatments, mHypoE-N46 cells were grown to 80–90% confluency and exposed either acutely, by treatment with CPF (1 pM) or DMSO vehicle for 4 h, or chronically, by treatment with CPF (1 pM) or DMSO vehicle for 6 days, with daily compound addition and re-plating on day 3. For antagonist experiments, in the acute condition cells were serum-starved for 16 h in serum-free DMEM (4.5 g/L glucose) containing 0.25% BSA and 1% penicillin/streptomycin, then pre-treated for 1 h with the ERβ antagonist PHTPP (10 µM) or the ERα antagonist MPP (10 µM), followed by CPF exposure (1 pM, 4 h). In the chronic condition, cells were exposed to CPF (1 pM) for 6 days, with daily addition of the compound and re-plating on day 3; on day 5, after 16 h of serum starvation in serum-free DMEM (4.5 g/L glucose, 0.25% BSA, 1% penicillin/streptomycin), cells were pre-treated for 1 h with PHTPP (10 µM) or MPP (10 µM), while CPF (1 pM) treatment was maintained. The Sulforhodamine B assay was performed as previously described (43) and optimized for toxicity screening of CPF (1 pM), DMSO vehicle, and puromycin (2 µg/mL) in 96-well plates. Following 4 h and 6 days of treatment, cells were fixed with cold 50% trichloroacetic acid (100 µL/well; final concentration 10%) for 1 h at 4°C, then stained with 0.4% SRB (Sigma-Aldrich) in 1% acetic acid (50 μL/well) for 30 min. Excess dye was removed by washing with 1% (v/v) acetic acid, and plates were air-dried. The protein-bound dye was subsequently solubilized in 100 mM Tris base solution, and absorbance was measured at 490 nm using a microplate reader.

### 2.2 Animals and hypothalamic tissue collection

All animal procedures were previously approved by the Ethical Committee of the Biogem Institute of Genetics Research “Gaetano Salvatore” (IRGS) and conducted in accordance with the European Council Directive 2010/63/EU and the Italian D. Lvo 26/14 (ID number 25-10). The present work did not involve new animal experimentation but relied on a secondary analysis of hypothalamic tissue collected in a previously published study (30). In that study, CD-1 mice were chronically exposed to CPF (10 mg/kg/day) from conception to 6 months of age while maintained on a standard diet. At postnatal day (PND) 180, animals were sacrificed by carbon dioxide inhalation, and blood and organs were collected. Hypothalami were dissected, snap-frozen in liquid nitrogen, and stored at -80°C until use. For the present analysis, hypothalami from CPF-treated (n = 5) and control (n = 5) male mice were homogenized using a TissueLyser LT (Qiagen, Hilden, Germany; RRID: SCR_020428) according to the manufacturer’s instructions.

### 2.3 RNA isolation and quantitative real-time PCR analysis

RNA extraction and purification from mHypoE-N46 cells and tissues was performed using QIAzol Lysis reagent (Qiagen, Hilden, Germany) according to the manufacturer’s protocol. For cDNA synthesis, 1 μg of total RNA was reverse transcribed using SuperScript™ III Reverse Transcriptase (Thermo Fisher Scientific). Quantitative real-time PCR (qPCR) was run in triplicate with iQ SYBR Green Supermix on an iCycler real-time detection system (Bio-Rad Laboratories) under the following cycling conditions: 95°C for 10 min, then 40 cycles of 95°C for 15 s and 60°C for 1 min. Each reaction (10 μL) contained 200 nM of each primer and 1X iQ SYBR Green Supermix, with 25 ng cDNA for cell samples and 10 ng cDNA for hypothalamic tissue. For *in vitro* experiments, relative quantification (RU) was determined by the 2^-ΔΔCt^ method. For *in vivo* analyses, absolute quantification (AU) of *Npy*, *Agrp*, *ERα*, *ERβ*, *Cart*, and *Pomc* mRNA levels used *Ribosomal Protein L7* (*Rpl7*) as the reference gene and the following standard curves: *Npy*, y = -4.2431x + 41.436; *Agrp*, y = -4.176x + 3.8478; *ERα*, y = -4.1936x + 42.124; *ERβ*, y = -3.486x + 35.542; *Cart*, y = -3.7863x + 39.506; *Pomc*, y = -3.3109x + 37.119; *Lepr*, y = -3.5278x + 36.417; *Rpl7*, y = –3.2877x + 11.796. Primers were designed with Primer-Blast and synthesized by Sigma-Aldrich. Primer sequences: *Npy* F, 5’-tggccagatacctactccgt-3’; *Npy* R, 5’-agggtcttcaagccttgttct-3’; *Agrp* F, 5’-gtgttctgctgttggcactg-3’; *Agrp* R, 5’-gatctagcacctccgccaaa-3’; *ERα* F, 5’-ggtgccctactacctggaga-3’; *ERα* R, 5’-gtctctctcggccattctgg-3’; *ERβ* F, 5’-agagagtagccggaagctga-3’; *ERβ* R, 5’-agaagcatcaggaggttggc-3’; *Cart* F, 5’-ctggacatctactctgccg-3’; *Cart* R, 5’-gtagatcggaatgcgtttactc-3’; *Pomc* F, 5’-caacctgctggcttgcatc-3’; *Pomc* R, 5’-cgtacttccgggggttttca-3’; *Lepr* F, 5’-accgaggaatcgttctgcaa-3’; *Lepr* R, 5’-gcagctatcacataaagaaattccc-3’; *Rpl7* F, 5’-aagcggattgccttgacaga-3’; *Rpl7* R, 5’-ttccttgaagcgtttcccga-3’.

### 2.4 Western blot analysis

WB analysis was performed as previously described (44). mHypoE-N46 cell lysates were prepared in RIPA buffer containing 20 mmol/L Tris–HCl (pH 7.5), 150 mmol/L NaCl, 10 mmol/L EDTA, 10 mmol/L Na_2_P_2_O_7_, 2 mmol/L Na_3_VO_4_, 100 mmol/L NaF, 1 mmol/L phenylmethylsulfonyl fluoride, and 10 μg/mL aprotinin (Sigma-Aldrich). Samples were incubated on ice for 30 min and clarified by centrifugation at 15,000 x g for 30 min at 4°C. Protein concentration was determined by Bradford assay (Bio-Rad Laboratories, Hercules, CA, USA; #5000001). Equal amounts of protein (60 μg) were separated by SDS-PAGE and transferred onto PVDF membranes. Membranes were incubated with primary antibodies against ERα (1:500; PA1-308, Thermo Fisher Scientific; RRID: AB_325813), ERβ (1:500; PA1-310B, Thermo Fisher Scientific; RRID: AB_325815), and vinculin (1:10,000; 7F9, sc-73614, Santa Cruz Biotechnology, Dallas, TX, USA; RRID: AB_1131294), followed by incubation with HRP-conjugated secondary antibodies (rabbit, 1:1000; #170-6515, Bio-Rad; RRID: AB_11125142; mouse, 1:10,000; #170-6516, Bio-Rad; RRID: AB_11125547). Immunoreactive bands were detected by chemiluminescence using ECL Star (GE Healthcare, Chicago, IL, USA) or SuperSignal West Pico PLUS (Thermo Fisher Scientific). Densitometric quantification was performed with ImageJ software (version 1.47t; RRID: SCR_003070).

### 2.5 ELISA assay

mHypoE-N46 cells were grown to 90% confluency. For acute treatments, cells were serum-starved for 16 h in serum-free DMEM (4.5 g/L glucose) supplemented with 0.25% BSA and 1% penicillin/streptomycin and then treated for 4 h with CPF (1 pM) or vehicle. For chronic treatments, cells were exposed daily to CPF (1 pM) or vehicle, with re-plating on day 3. On day 5, cells were serum-starved for 16 h under the same conditions, while maintaining CPF or vehicle exposure. Cell suspensions were collected and concentrated using C18 SEP-Columns (Phoenix Pharmaceuticals, Burlingame, California, USA) and SpeedVac vacuum concentrators (ECO Vide, Rome, Italy). Npy and Agrp concentrations were determined by enzyme-linked immunosorbent assays (ELISAs; Phoenix Pharmaceuticals) according to the manufacturer’s protocols (sensitivity: 0.09 ng/mL). Data analysis and concentration calculations were performed using MyAssays software (www.myassays.com; RRID: SCR_016562).

### 2.6 Statistical procedures

For each investigation, experiments were performed under identical conditions, using the same reagent batches, instruments, and investigator. Technical and biological replicates were included to exclude experimental errors and biological variability. Unless otherwise specified, three independent experiments were conducted for *in vitro* data and at least five animals were analyzed for *in vivo* experiments. Fold-change (FC) values were calculated as the ratio between mean results in treated and control samples. The Shapiro-Wilk test was used to assess normality of data distribution. For symmetrically distributed variables, results are presented as mean ± SD, and group comparisons were performed using unpaired two-tailed Student’s t-test or one-way ANOVA followed by Tukey multiple comparisons test, as appropriate. For non-normally distributed variables, data are presented as median [Q1-Q3], and comparisons were performed using the unpaired two-tailed Student’s t-test. Correlations were assessed using Pearson’s correlation test for normally distributed variables. A p-value < 0.05 was considered statistically significant. Statistical analyses were performed using GraphPad Prism version 8.4.2 (GraphPad Software Inc., La Jolla, CA, USA; RRID: SCR_000306).

## 3 Results

### 3.1 CPF exposure, both acute and chronic, up-regulates orexigenic gene expression and neuropeptide secretion in mHypoE-N46 cells

**Figure 1:**
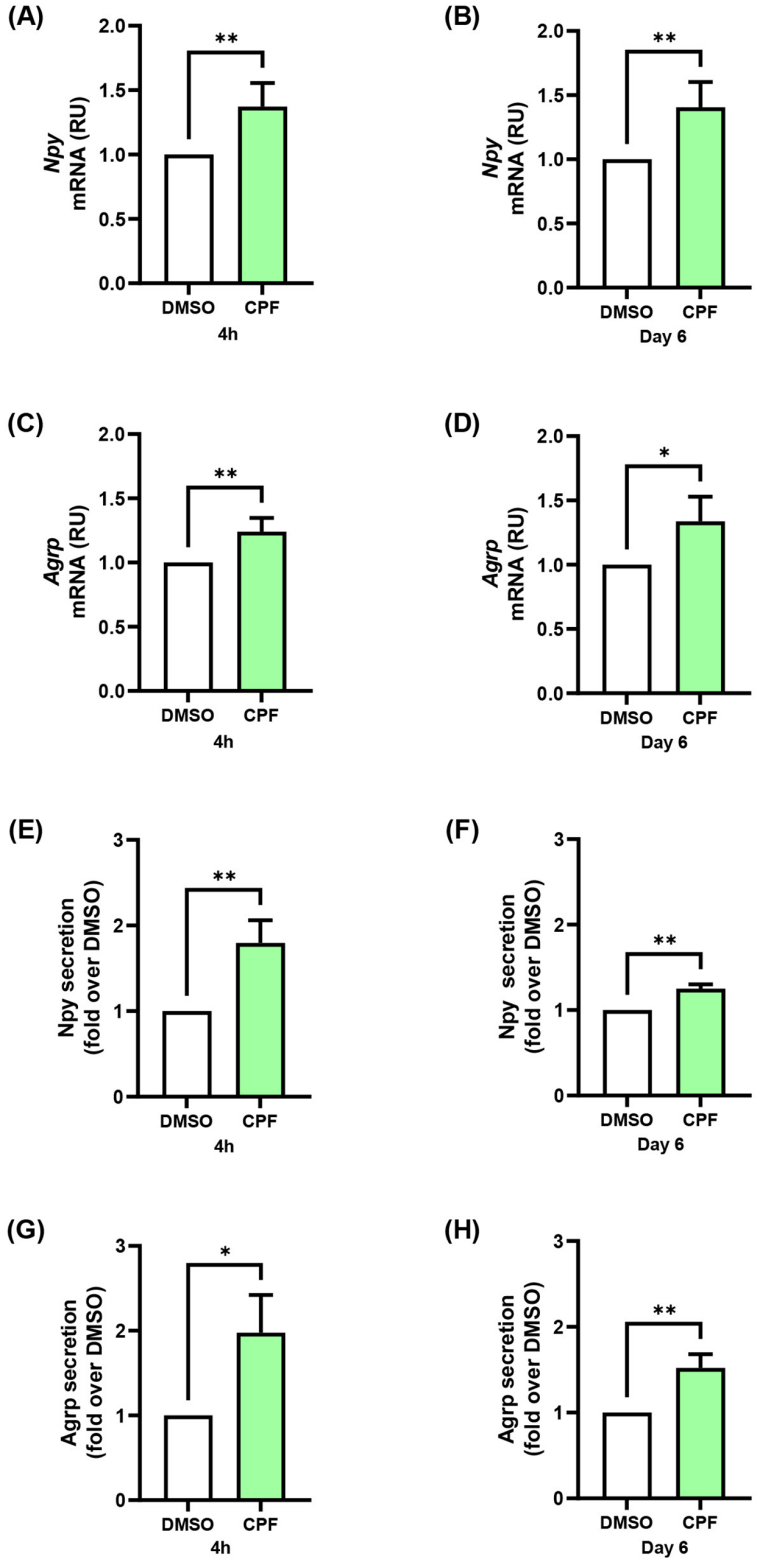
Effects of acute (4 h) and chronic (day 6) CPF exposure on orexigenic gene expression and basal secretion in mHypoE-N46 cells. **(A, B)***Npy* and **(C, D)***Agrp* mRNA levels were quantified by qPCR in cells treated with CPF (1 pM) for 4 h or 6 days and compared with vehicle-treated controls (DMSO). qPCR data are presented as relative units (RU) over vehicle and shown as mean ± SD from four independent experiments performed in triplicate. Statistical significance was assessed using an unpaired two-tailed Student’s t-test (*p < 0.05, **p < 0.01 vs. DMSO). **(E, F)** Npy and **(G, H)** Agrp concentrations were measured in conditioned medium (CM) from cells treated with CPF (1 pM) for 4 h or 6 days and compared with vehicle-treated controls (DMSO). Neuropeptide concentrations were determined by ELISA (ng/mL). ELISA data are shown as mean ± SD from three independent experiments performed in triplicate. Statistical significance was assessed using an unpaired two-tailed Student’s t-test (*p < 0.05, **p < 0.01 vs. DMSO).

To investigate the direct impact of CPF on hypothalamic orexigenic pathways, we used the murine clonal hypothalamic cell line mHypoE-N46, an established in vitro model that robustly expresses Npy and Agrp (45). For all experiments, we selected a 1 pM CPF dose, as it closely corresponds to CPF concentrations detected in humans (32, 46). At this dose, CPF did not alter cell viability, as determined by the Sulforhodamine B assay (**Supplementary Figure 1**), under either of the two exposure times selected to evaluate both acute (4 h) and chronic (6 day) effects on orexigenic pathway regulation. CPF significantly increased the expression of both orexigenic genes under acute (4 h) and chronic (6 day) conditions (**Figures 1A–D**). Npy mRNA levels were up-regulated 1.4-fold in both conditions (p < 0.01), whereas Agrp mRNA rose by 1.2-fold (p < 0.01) and 1.3-fold (p < 0.05), respectively, compared with the vehicle. These transcriptional changes were paralleled by increased neuropeptide release into conditioned media (CM) from mHypoE-N46 under acute and chronic CPF treatments (**Figures 1E–H**). Basal Npy secretion, indeed, rose by 1.8-fold (p < 0.01) in acute and 1.3-fold (p < 0.01) in chronic exposure, while basal Agrp secretion increased by 2.0-fold (p < 0.05) and 1.5-fold (p < 0.01), respectively. Altogether, these results demonstrate that short- and long-term CPF exposure enhances the expression and secretion of key orexigenic neuropeptides in hypothalamic neurons.

### 3.2 CPF exposure, both acute and chronic, enhances orexigenic signaling in hypothalamic neurons through ERβ-dependent mechanisms

**Figure 2:**
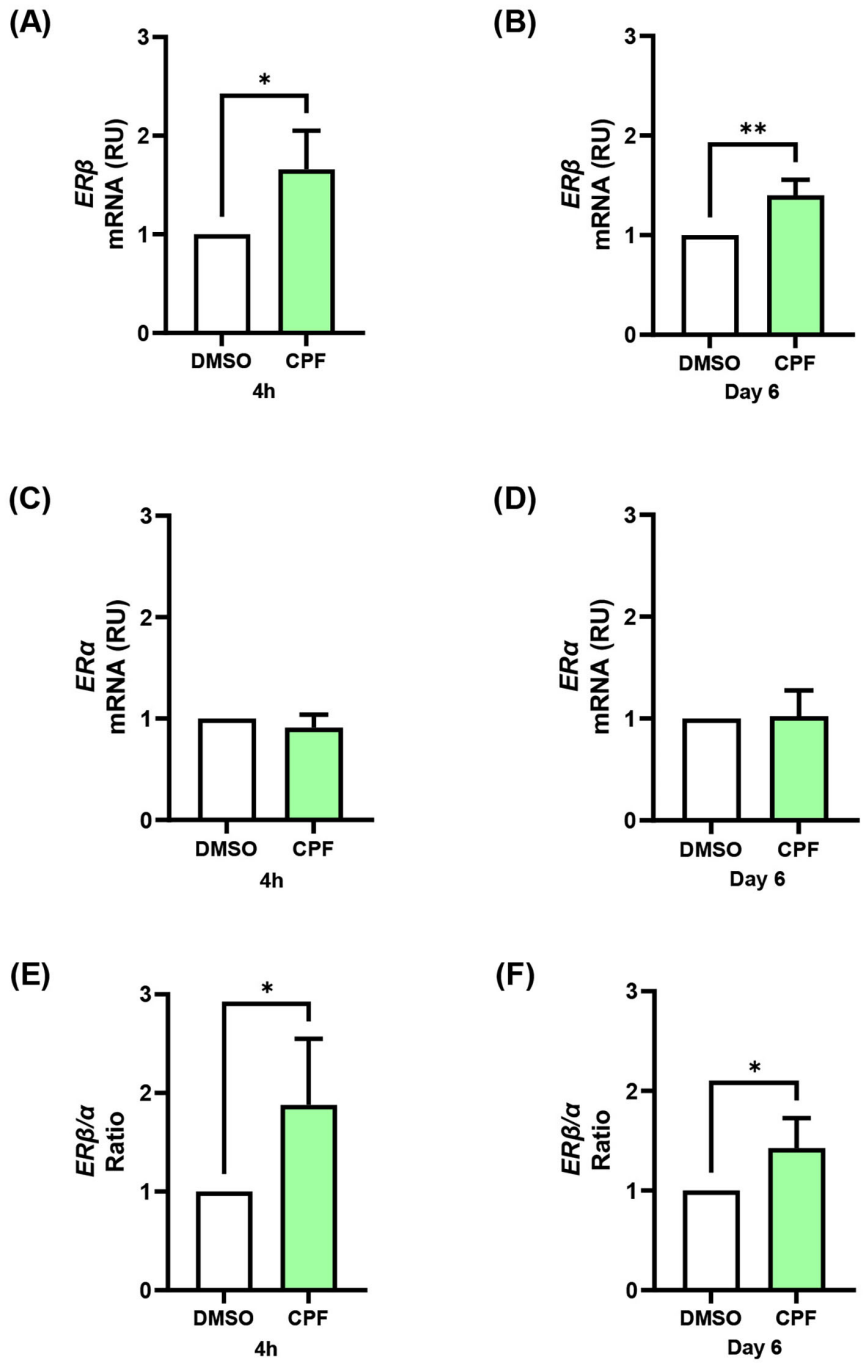
Effects of acute (4 h) and chronic (day 6) CPF exposure on estrogen receptor gene expression in mHypoE-N46 cells. **(A, B)***ERβ* and **(C, D)***ERα* mRNA levels, and **(E, F)***ERβ*/*ERα* expression ratio, were quantified by qPCR in cells treated with CPF (1 pM) for 4 h or 6 days and compared with vehicle-treated controls (DMSO). Data are expressed as relative units (RU) over vehicle and shown as mean ± SD four independent experiments performed in triplicate. Statistical significance was assessed using unpaired two-tailed Student’s *t*-test (*p < 0.05, **p < 0.01 vs. DMSO).

**Figure 3:**
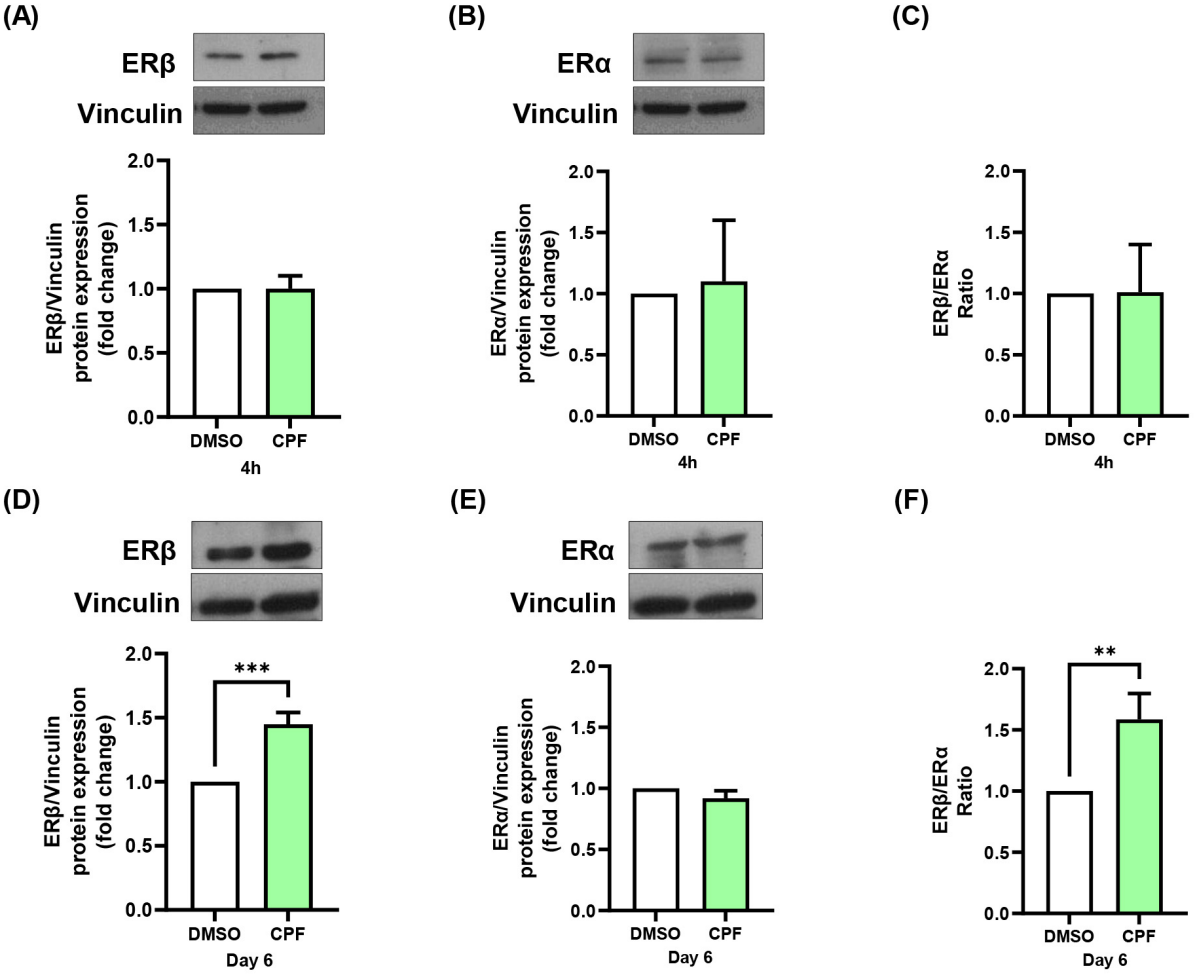
Effects of acute (4 h) and chronic (day 6) CPF exposure on estrogen receptor protein levels in mHypoE-N46 cells. ERβ and ERα protein levels were assessed by immunoblotting in cells treated with CPF (1 pM) for 4 h or 6 days and compared with vehicle-treated controls (DMSO). Densitometric analysis of ERβ **(A)** and ERα **(B)** protein levels and **(C)** ERβ/ERα protein ratio after 4 h treatment. Densitometric analysis of ERβ **(D)** and ERα **(E)** protein levels and **(F)** ERβ/ERα protein ratio after 6 days of treatment. Vinculin was used as loading control. Densitometry data are expressed as fold changes relative to vehicle-treated controls (DMSO) and shown as mean ± SD from three independent experiments. Statistical significance was assessed using unpaired two-tailed Student’s *t*-test (**p < 0.01, ***p < 0.001 vs. DMSO).

**Figure 4:**
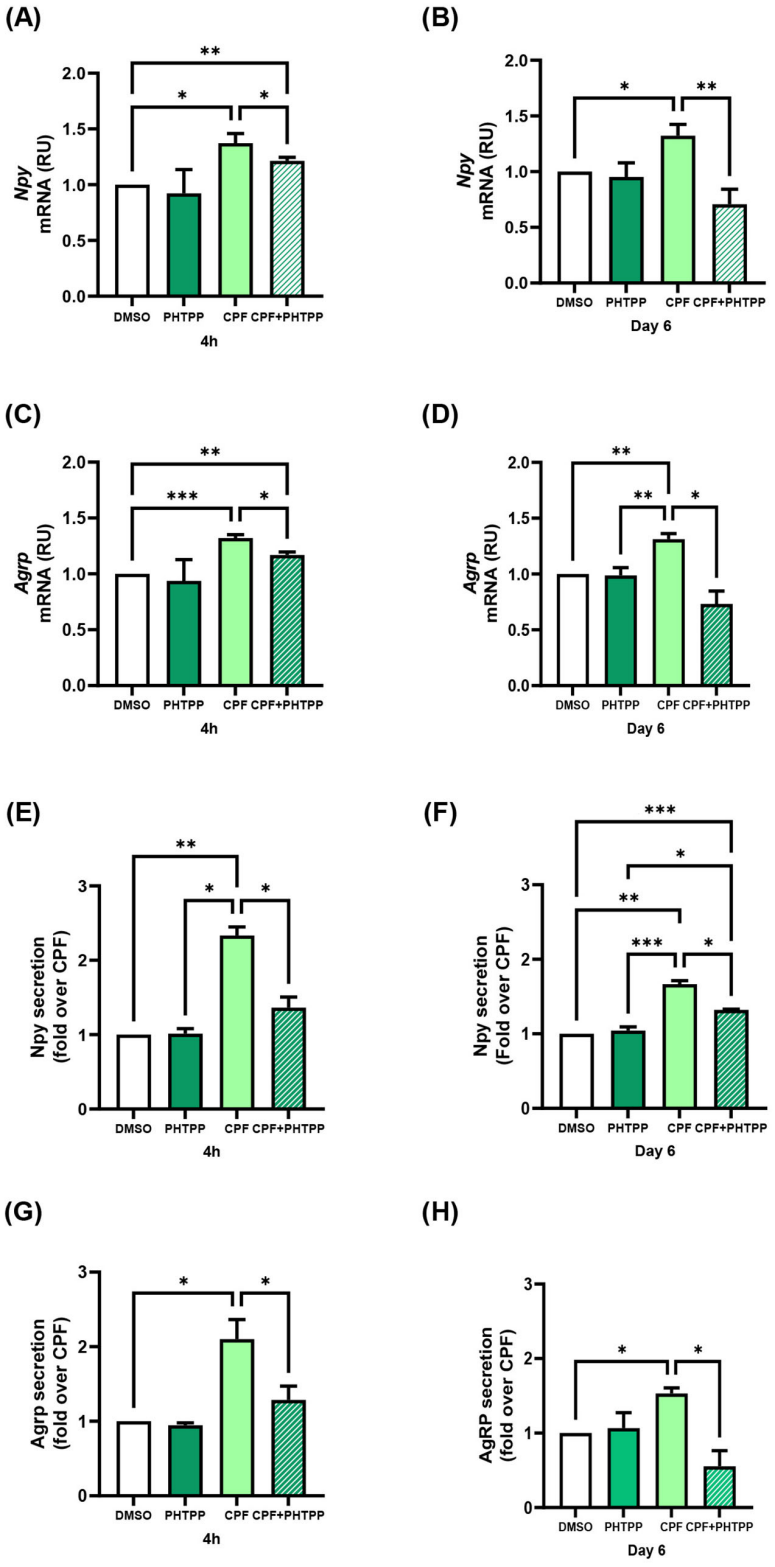
Effects of ERβ antagonist (PHTPP) on CPF-induced orexigenic gene expression and basal secretion in mHypoE-N46 cells. **(A, B)***Npy* and **(C, D)***Agrp* mRNA levels were quantified by qPCR in cells treated with CPF (1 pM) alone, ERβ antagonist PHTPP (10µM) alone or CPF in combination with the PHTPP for 4 h or 6 days and compared with vehicle-treated controls (DMSO). qPCR data are presented as relative units (RU) over vehicle and shown as mean ± SD from four independent experiments performed in triplicate. Statistical significance was assessed using one-way ANOVA followed by Tukey multiple comparisons test (*p < 0.05, **p < 0.01, ***p < 0.001). **(E, F)** Npy and **(G, H)** Agrp concentrations were measured in conditioned medium (CM) from cells treated with CPF (1 pM) alone, PHTPP (10µM) alone or CPF in combination with PHTPP for 4 h or 6 days and compared with vehicle-treated controls (DMSO). Neuropeptide levels were quantified by ELISA (ng/mL), expressed as fold changes relative to vehicle-treated controls (DMSO). ELISA data are shown as mean ± SD from three independent experiments performed in triplicate. Statistical significance was assessed using one-way ANOVA followed by Tukey multiple comparisons test (*p < 0.05).

**Figure 5:**
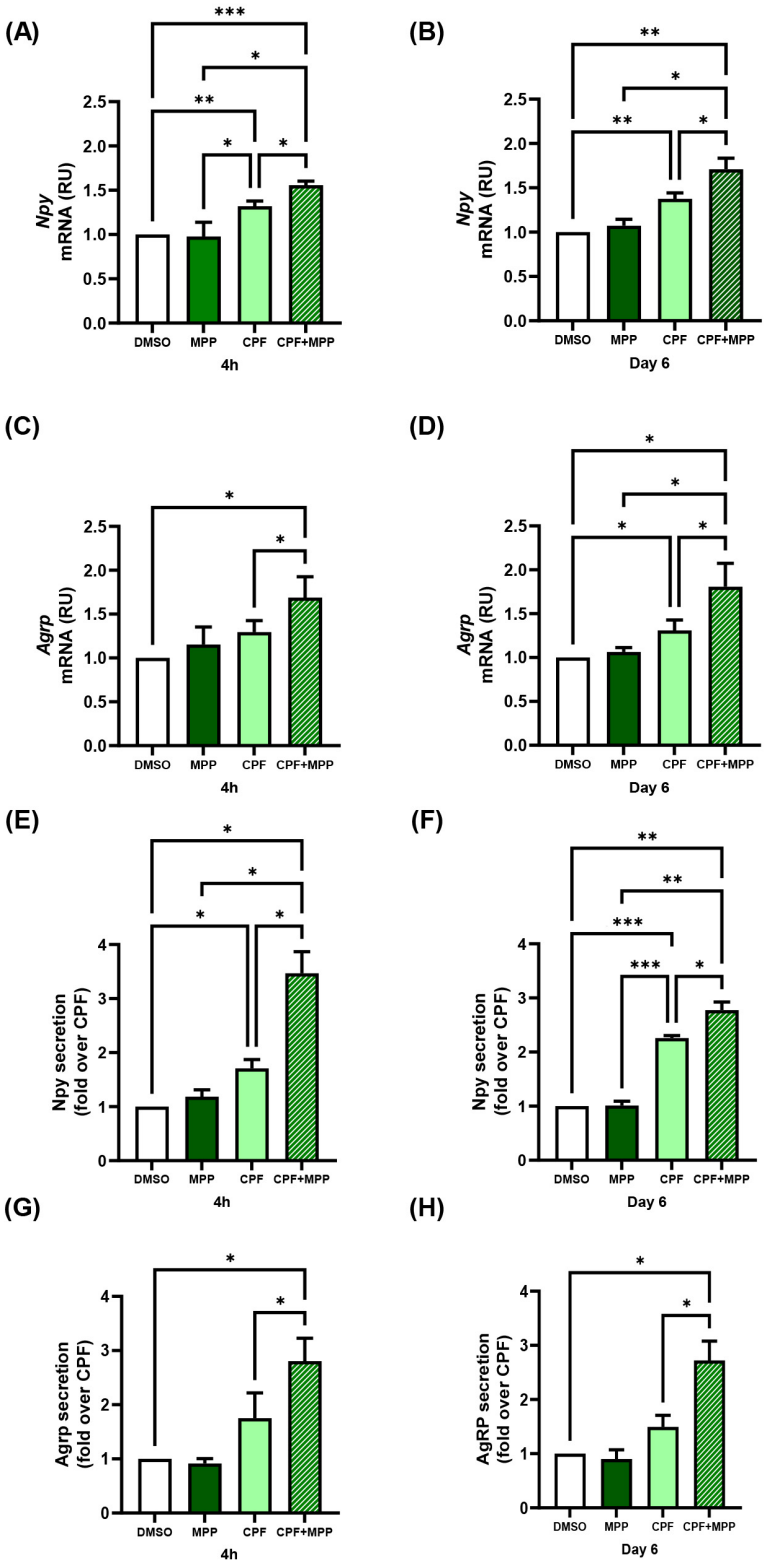
Effects of ERα antagonist (MPP) on CPF-induced orexigenic gene expression and basal secretion in mHypoE-N46 cells. **(A, B)** Npy and **(C, D)** Agrp mRNA levels were quantified by qPCR in cells treated with CPF (1 pM) alone, ERα antagonist MPP (10 µM) alone or CPF in combination with MPP for 4 h or 6 days and compared with vehicle-treated controls (DMSO). qPCR data are presented as relative units (RU) over vehicle and shown as mean ± SD from four independent experiments performed in triplicate. Statistical significance was assessed using one-way ANOVA followed by Tukey multiple comparisons test (*p < 0.05, **p < 0.01, ***p < 0.001 vs. DMSO). **(E, F)** Npy and **(G, H)** Agrp concentrations were measured in conditioned medium (CM) from cells treated with CPF (1 pM) alone, MPP (10 µM) alone or CPF in combination with MPP for 4 h or 6 days and compared with vehicle-treated controls (DMSO). Neuropeptide levels were quantified by ELISA (ng/mL), expressed as fold changes relative to vehicle-treated controls (DMSO). ELISA data are shown as mean ± SD from three independent experiments performed in triplicate. Statistical significance was assessed using one-way ANOVA followed by Tukey multiple comparisons test (*p < 0.05, **p < 0.01, ***p < 0.001 vs. DMSO).

CPF has been reported to exert estrogen-like effects in other cellular contexts (47–49). We thus investigated whether it modulates ERα and ERβ expression in mHypoE-N46 hypothalamic cells and whether such changes contribute to the regulation of orexigenic neuropeptides. Indeed, estrogen is known to regulate Npy and Agrp expression in hypothalamic neurons through modulation of the ERα/ERβ ratio (50). To address this issue, we measured ERα and ERβ gene expression in CPF-treated mHypoE-N46 cells under acute (4 h) and chronic (6 day) exposure. ERβ mRNA increased by 1.4-fold in both conditions (p < 0.05 and p < 0.01, respectively; **Figures 2A, B**), whereas ERα mRNA remained unchanged (**Figures 2C, D**). As a result, the ERβ/ERα mRNA ratio rose by 1.9-fold (p < 0.05) after acute treatment and by 1.4-fold (p < 0.05) after chronic treatment (**Figures 2E, F**). At the protein level, acute CPF exposure did not significantly affect ERβ or ERα abundance, nor their ratio (**Figures 3A–C**). In contrast, chronic exposure led to a 1.5-fold increase in ERβ protein (p < 0.01), with no change in ERα, resulting in a 1.6-fold increase in the ERβ/ERα protein ratio (p < 0.01) (**Figures 3D–F**). These data indicate that CPF can shift ER signaling toward ERβ dominance, although functional estrogen-like effects may also occur via activation of pre-existing receptor pools, particularly in the acute phase. To directly test whether ERβ mediates CPF-induced orexigenic gene regulation, we used ER-selective antagonists. As previously reported in **Figure 1** and further confirmed in **Figures 4**, **5**, CPF alone significantly enhanced Npy and Agrp expression and secretion. Importantly, neither PHTPP nor MPP alone modified basal Npy or Agrp mRNA levels or secretion (**Figures 4**, **5**). Co-treatment with the ERβ-selective antagonist PHTPP markedly blunted CPF-induced Npy and Agrp upregulation. Acute co-exposure reduced Npy and Agrp mRNA by 11.3% (p < 0.05; **Figure 4A**) and 11.4% (p < 0.05; **Figure 4C**), respectively, while chronic co-exposure decreased them by 46.7% (p < 0.01; **Figure 4B**) and 43.8% (p < 0.05; **Figure 4D**). Similarly, basal Npy and Agrp secretion into CM from mHypoE-N46 cells was reduced under both acute (-41.6% and -39.5%, p < 0.05 and p < 0.05; **Figures 4E, G**) and chronic (-20.8% and -64.0%, p < 0.05 and p < 0.05; **Figures 4F, H**) CPF treatments. Conversely, inhibition of ERα with MPP enhanced CPF effects. Acute co-treatment with MPP and CPF increased Npy mRNA by 1.2-fold (p < 0.05; **Figure 5A**) and Agrp mRNA by 1.3-fold (p < 0.05; **Figure 5C**) compared with CPF alone. Also, in chronic co-treatment, Npy and Agrp mRNA levels rose by 1.2-fold (p < 0.05**;****Figure 5B**) and 1.4-fold (p < 0.05; **Figure 5D**), respectively. Basal neuropeptide secretion also increased. Npy and Agrp levels in CM from mHypoE-N46 cells were elevated by 2.0-fold (p < 0.05) and 1.6-fold (p < 0.05) under acute conditions (**Figures 5E, G**), and by 1.2-fold (p < 0.05) and 2.3-fold (p < 0.05) under chronic exposure (**Figures 5F, H**). Together, these results suggest that both acute and chronic CPF exposure may enhance orexigenic signaling in hypothalamic neurons, at least in part via ERβ activation. This effect could involve a combination of rapid, non-genomic actions on pre-existing ERβ and longer-term transcriptional changes that shift the ERα/ERβ balance toward ERβ dominance.

### 3.3 Six-month CPF exposure in CD-1 mice increases hypothalamic Npy/Agrp expression and ERβ levels

**Figure 6:**
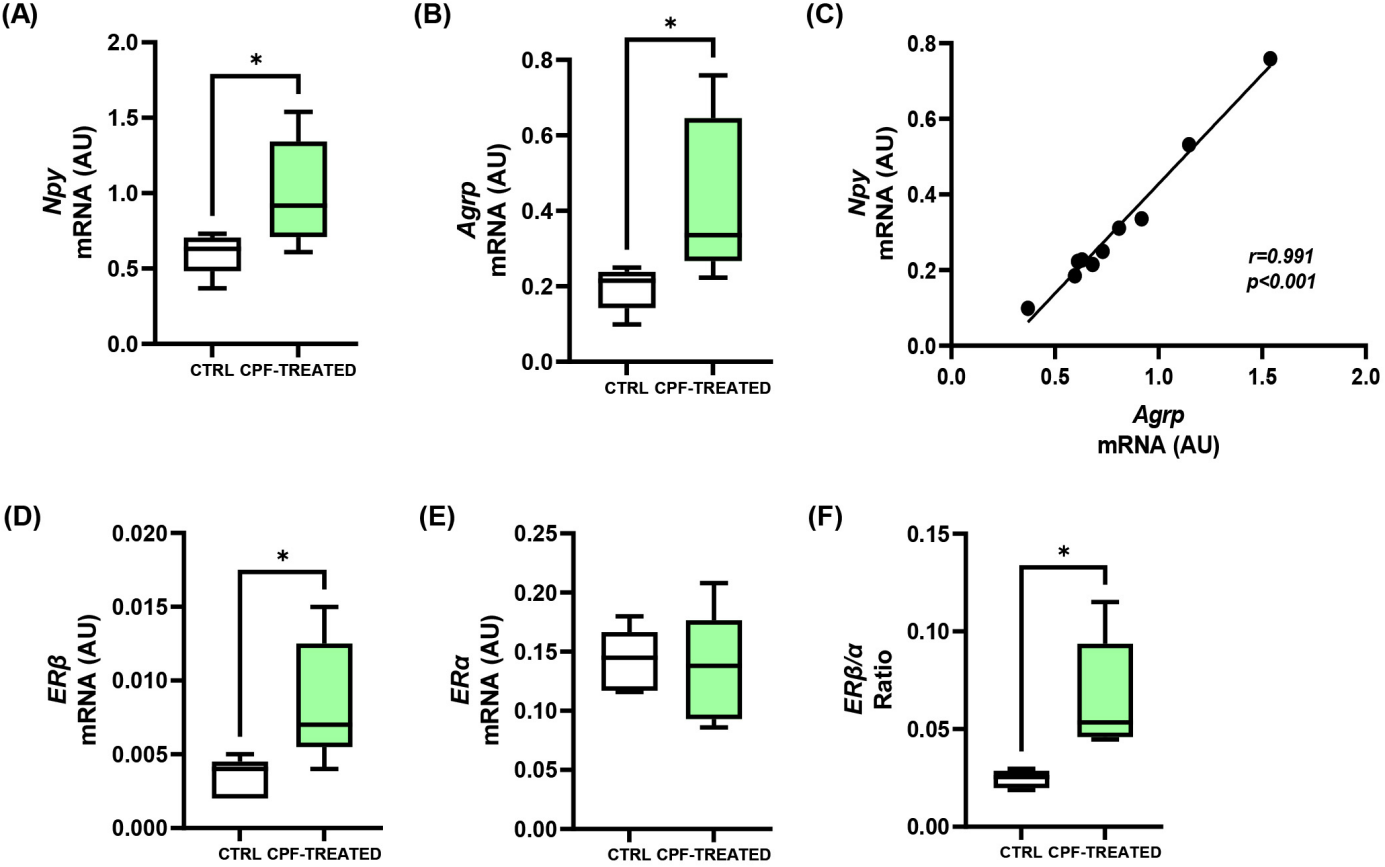
Effects of CPF exposure on hypothalamic orexigenic and estrogen receptor gene expression in mice. Hypothalami were collected from CD-1 mice chronically exposed to CPF (10 mg/kg/day) from conception to 6 months of age while maintained on a standard diet. **(A, B)***Npy* and *Agrp* mRNA levels, and **(C)** correlation analysis between *Npy* and *Agrp* expression. **(D, E)***ERβ* and *ERα* mRNA levels, and **(F)***ERβ*/*ERα* expression ratio. Total RNA was analyzed by qPCR. Data are expressed as absolute units (AU) and presented as boxplots showing mean values from *n* = 5 mice per group. Normality was assessed using the Shapiro-Wilk test. Statistical differences were evaluated using unpaired two-tailed Student’s *t*-test (*p < 0.05 vs. CTRL). Pearson’s correlation coefficient (*r*) and p-value are indicated.

To provide in vivo support for a subset of the in vitro observations, we performed a secondary analysis of hypothalamic tissue collected from a previously published animal study (30). In that work, CD-1 mice were chronically exposed to CPF (10 mg/kg/day) from conception to 6 months of age while maintained on a standard diet. In male mice, CPF exposure significantly affected the gene expression of hypothalamic orexigenic neuropeptides (**Figures 6A–C**). Indeed, the mRNA levels of Npy and Agrp were increased by 1.7-fold (p < 0.05; **Figure 6A**) and by 2.2-fold (p < 0.05; **Figure 6B**), respectively, in CPF-treated CD-1 mice compared with unexposed controls. A strong positive correlation was observed between Npy and Agrp expression (n = 10; r = 0.991; p < 0.001; **Figure 6C**). In contrast, no significant differences were detected for the anorexigenic neuropeptides Pomc (**Supplementary Figure 2A**) and Cart (**Supplementary Figure 2B**). In line with the in vitro findings, CPF treatment also increased ERβ transcript levels by 2.4-fold (p < 0.05; **Figure 6D**) in CPF-treated CD-1 mice compared with unexposed controls, without affecting ERα expression (**Figure 6E**). Consequently, the ERβ/ERα ratio rose by 2.7-fold (p < 0.05; **Figure 6F**). These findings indicate that CPF selectively targets the orexigenic arm of hypothalamic neuropeptide regulation and support the involvement of ERβ in mediating this effect in vivo, thereby providing physiological confirmation of the mechanistic insights obtained in vitro.

### 3.4 CPF exposure increases Lepr expression both *in vitro* and *in vivo*

**Figure 7:**
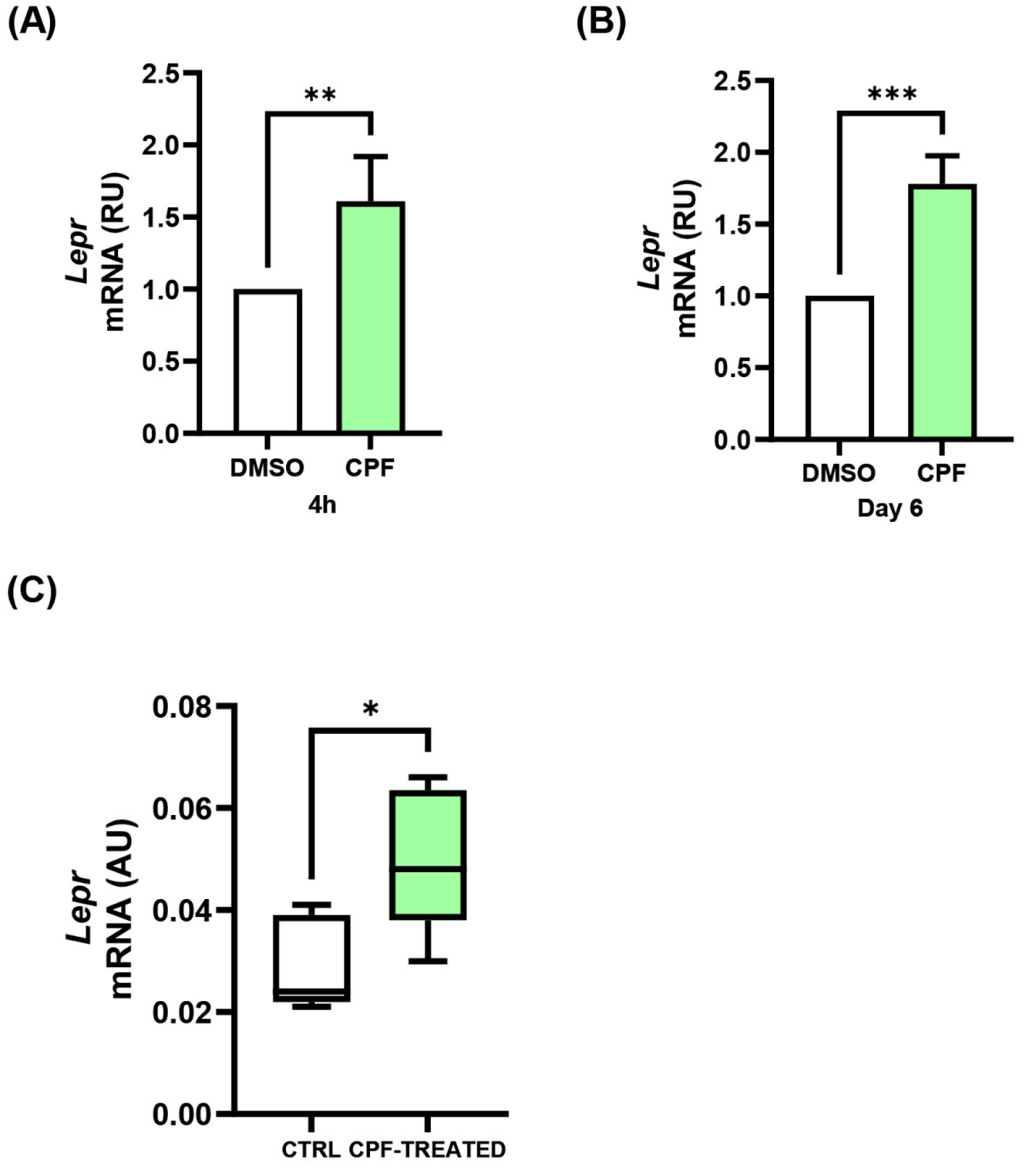
Effects of acute (4 h) and chronic (day 6) CPF exposure on *leptin receptor* (*Lepr*) expression in mHypoE-N46 cells and *in vivo* hypothalamic tissue. **(A, B)***Lepr* mRNA levels were quantified by qPCR in mHypoE-N46 cells treated with CPF (1 pM) for 4 h **(A)** or 6 days **(B)** and compared with vehicle-treated controls (DMSO). Data are expressed as relative units (RU) over vehicle and shown as mean ± SD four independent experiments performed in triplicate. Statistical significance was assessed using unpaired two-tailed Student’s t-test (**p < 0.01, ***p < 0.001 vs. DMSO). **(C)***Lepr* mRNA expression was analyzed by qPCR in hypothalamic tissue from 6-month-old male CD-1 mice chronically exposed to CPF (10 mg/kg/day) from conception to sacrifice while maintained on a standard diet. Data are expressed as absolute units (AU) and presented as boxplots showing mean values from n = 5 mice per group. Normality was assessed using the Shapiro-Wilk test. Statistical differences were evaluated using unpaired two-tailed Student’s t-test (*p < 0.05 vs. CTRL).

We next investigated whether the dysregulating effects of CPF might extend to Lepr expression, an upstream homeostatic regulator of NPY/AgRP neuronal activity whose expression has been reported to be modulated under different metabolic conditions (51–54). In mHypoE-N46 cells, CPF significantly increased Lepr mRNA levels under both acute (4 h) and chronic (6 day) exposure (**Figures 7A, B**). Under acute conditions, Lepr expression was up-regulated by 1.6-fold compared with vehicle-treated controls (p < 0.01; **Figure 7A**). Similarly, chronic CPF exposure resulted in a 1.8-fold increase in Lepr mRNA levels relative to controls (p < 0.001; **Figure 7B**). Consistently, increased Lepr expression was also detected in hypothalamic samples from CPF-exposed mice compared with control animals, corresponding to a 1.7-fold up-regulation in CPF-treated mice (p < 0.05; **Figure 7C**). These findings indicate that CPF exposure enhances Lepr expression across experimental models, indicating that CPF may also intersect with leptin-related regulatory mechanisms.

## 4 Discussion

The organophosphate pesticide CPF is widely recognized for its neurotoxicity (19, 20). However, beyond cholinesterase inhibition, a growing body of evidence implicates CPF in metabolic disruption (29, 30), and animal studies indicate that even low-dose exposure can promote obesity (31, 32). While the peripheral mechanisms underlying CPF-induced obesity have been partly elucidated (32), it remains unclear whether, and in what manner, CPF may affect the central regulation of energy homeostasis.

In this study, we examined the effects of CPF on hypothalamic neuropeptides using the murine embryonic hypothalamic cell line mHypoE-N46, an extensively used clonal model for investigating orexigenic neuropeptide regulation, as these cells endogenously express *Npy* and *Agrp* and respond to metabolic cues (45). Cells were exposed to CPF at a concentration of 1 pM, a dose confirmed not to affect cell viability in our model. This concentration is well below those typically associated with acetylcholinesterase inhibition or toxicity and provides a realistic approximation of human exposure levels (32, 46). Although many studies have focused on the effects of CPF at toxic or high doses, increasing attention has been directed toward the potential metabolic consequences of low-dose exposure (30, 32). In addition, both acute (4 h) and chronic (6-day) *in vitro* treatments at 1 pM CPF were applied to explore potential human exposure patterns. Acute low-dose exposure allows the evaluation of immediate cellular responses that may follow transient peaks of CPF intake or inhalation. In contrast, chronic low-dose exposure better reflects the sustained and cumulative nature of dietary environmental exposure, providing insight into subtle metabolic effects that may emerge over time. Under these conditions, both acute and chronic CPF exposure significantly increased *Npy* and *Agrp* mRNA expression and secretion above basal levels, demonstrating that CPF can directly modulate orexigenic pathways in hypothalamic neurons.

Estrogens regulate key metabolic processes, including food intake and fat distribution (55–57). Estrogenic mechanisms are also exploited by several well-known endocrine disruptors, such as BPA, which interacts with ERα and ERβ to modulate metabolic and neuronal pathways (58, 59). Emerging evidence indicates that CPF may also interfere with estrogenic signaling (47–49). Here we found that, in mHypoE-N46 hypothalamic cells, CPF treatment increased *ERβ* mRNA under both acute and chronic conditions, while *ERα* expression remained unchanged. This resulted in an increased *ERβ*/*ERα* ratio, a shift in stoichiometry that may alter dimer composition toward ERβ homodimers or ERβ/ERα heterodimers, thereby influencing target gene regulation. Notably, Titolo et al. demonstrated in the mHypoE-N38 that the expression of *Npy* and *Agrp* is directly regulated by estrogen in a biphasic manner, tightly linked to dynamic changes in ER stoichiometry (50). In particular, they showed that a shift toward ERβ dominance favors ERβ-driven signaling, underlying the induction of orexigenic neuropeptides. Our data are consistent with this model, indicating that CPF may promote ERβ-dependent transcriptional control of *Npy* and *Agrp*.

At the protein level, only chronic CPF treatment elevated ERβ, likely reflecting a temporal dissociation between transcription and translation. Such a delay may be due to mechanisms involving translational efficiency, mRNA stability, or protein turnover (60, 61). Interestingly, despite the absence of ERβ protein induction at 4 hours, CPF was sufficient to increase basal Npy and Agrp secretion acutely. Co-treatment with the ERβ-selective antagonist PHTPP significantly reduced Npy and Agrp mRNA expression and secretion compared to CPF alone, under both acute and chronic conditions. This finding indicates that CPF acts through ERβ, even in the absence of detectable protein upregulation at early time points, consistent with activation of pre-existing ERβ pools. Indeed, ERβ is known to trigger rapid, ligand-dependent, extranuclear signaling, among which are the MAPK and PI3K/Akt pathways (62) and is detectable in membrane-associated complexes (63). These observations support a two-phase model: an early response mediated by membrane-bound ERβ, followed by a later transcription-dependent phase requiring *de novo* protein synthesis. However, additional studies will be needed to establish whether CPF activates ERβ-dependent downstream signaling cascades in hypothalamic neurons. Importantly, selective inhibition of ERα with MPP further increased Npy and Agrp mRNA levels and secretion. This is in agreement with previous evidence suggesting that ERα can exert an inhibitory or modulatory influence on orexigenic signaling, thereby counterbalancing the stimulatory role of ERβ (50). Altogether, these findings indicate that while ERβ represents the primary mediator of CPF action in this context, ERα may exert opposing or modulatory effects.

An additional finding of this study is that, *in vitro*, both acute and chronic CPF exposure led to a significant increase in *Lepr* expression. This aligns with reports of increased *Lepr* expression following exposure to the endocrine disruptor BPA (51, 52). Notably, increased *Lepr* mRNA has also been described in leptin-deficient obese ob/ob mice models, particularly within the arcuate and ventromedial hypothalamic nuclei, where it is interpreted as a compensatory response to the absence of leptin binding and impaired leptin signaling (53, 54). Furthermore, Mercer et al. reported that hypothalamic *Lepr* expression is up-regulated in metabolic states associated with negative energy balance, such cold-exposed lean mice (54). Together, these findings support the hypothesis that CPF may interfere with hypothalamic leptin sensitivity, underscoring the need for further investigation of leptin signaling under CPF exposure.

Finally, here we report that CD-1 male mice chronically exposed to CPF (10 mg/kg/day) from conception to 6 months of age exhibited increased hypothalamic mRNA expression of *Npy* and *Agrp*, closely mirroring the *in vitro* findings. A strong positive correlation between the two neuropeptides was observed, indicating co-regulation in response to CPF. Importantly, no significant changes were detected in the anorexigenic neuropeptides *Pomc* and *Cart*, supporting a selective effect on appetite-promoting pathways. Moreover, CPF exposure significantly increased *ERβ* mRNA expression and the *ERβ*/*ERα* ratio in the hypothalamus, without altering *ERα* expression. These *in vivo* observations further support ERβ as a key mediator of CPF-induced hypothalamic dysfunction. Furthermore, *Lepr* mRNA was up-regulated in the hypothalamus of CPF-exposed mice, supporting the relevance of this response *in vivo*. In line with the central molecular alterations identified in the present study, previous work using the same CD-1 cohort demonstrated a modest but persistent upward trend in body weight under standard diet conditions in males, but not in females (30). Furthermore, preliminary observations from a related study currently under revision by our group indicate that this sex-specific phenotypic divergence emerges earlier in life, with CPF-exposed males, but not females, displaying an accelerated post-weaning body weight trajectory followed by a persistent upward trend under the same dietary conditions. Although modest in magnitude under standard diet conditions, these subtle phenotypic changes may reflect an acquired susceptibility rather than the absence of effect, consistent with a model in which CPF induces initial metabolic perturbations that can be subsequently compensated over time in non-obesogenic contexts. This interpretation aligns with emerging evidence indicating that the metabolic consequences of CPF are conditionally expressed rather than uniformly manifested (31, 32). For instance, Wang et al. reported that in C57BL/6J male mice, CPF exposure initiated in adulthood did not alter body weight or fat mass when animals were maintained on a control diet, whereas a marked obesogenic phenotype emerged only when CPF was combined with a high-fat diet under thermoneutral conditions, resulting in increased adiposity and impaired glucose metabolism (32). Likewise, Peris-Sampedro et al. demonstrated that the obesogenic response to CPF appears to depend on genetic susceptibility, as significant weight gain and metabolic impairment were observed only in mice carrying the human *APOE3* genotype, whereas wild-type C57BL/6N mice exhibited only nonsignificant trends under identical exposure conditions (31). Together, these findings support the view that CPF may act as a latent metabolic disruptor, engaging mechanisms of metabolic regulation, including hypothalamic molecular reprogramming, that may predispose to increased vulnerability when combined with additional environmental or genetic stressors.

A limitation of our work is that the *in vivo* findings derive from a secondary analysis of a previous study (30), which limited our ability to evaluate mechanistic aspects explored *in vitro* and to directly assess CPF in the context of an obesogenic diet. A further limitation is that the *in vivo* analyses were performed exclusively in male mice. As discussed above, hypothalamic energy regulation is profoundly influenced by sex hormones (55–57), and estrogen signaling engages ERα and ERβ in a sex-dependent manner, with ERα generally exerting anorexigenic and ERβ more orexigenic influences on NPY/AgRP circuitry both *in vitro* and *in vivo* (50, 64). Moreover, leptin sensitivity is modulated by the hormonal milieu, with estrogen playing a key role in enhancing hypothalamic leptin responsiveness (65–67) Consequently, the CPF-driven shift toward ERβ signaling observed in males may not necessarily occur in females, where higher or fluctuating estrogen levels could modify the hypothalamic response (66, 67). Consistent with this notion, and as previously mentioned, in the same CPF exposure model CD-1 female mice did not display differences in body weight compared with controls at six months of age (30), suggesting that the metabolic phenotype associated with CPF may differ between sexes. Unfortunately, the lack of available hypothalamic samples from CPF-exposed female mice did not allow us in the present study to evaluate whether the molecular alterations identified in males are also present in females. This limitation also prevented the assessment of potential sex-dependent mechanisms underlying such phenotypic divergence. Future studies specifically designed to integrate CPF exposure with high-fat or high-sugar diets and to include both male and female mice will be essential to determine whether CPF exacerbates diet-induced obesity and metabolic impairment, and to elucidate potential sex-dependent differences in hypothalamic responsiveness to CPF.

## 5 Conclusions

In conclusion, our findings demonstrate that CPF acts as a central endocrine disruptor by selectively engaging ERβ, thereby enhancing the expression and secretion of the orexigenic neuropeptides Npy and Agrp. By shifting the ERβ/ERα balance toward ERβ-driven signaling, CPF may function as an ERβ-selective modulator with the potential to influence energy homeostasis and feeding behavior. In addition, CPF induced transcriptional changes in *Lepr*, possibly reflecting adaptive modulation within hypothalamic regulatory circuits. These results highlight CPF as a metabolic disruptor acting through central regulatory pathways. Given the evolutionary conservation of hypothalamic circuits, which implies that similar mechanisms may occur across species, including humans, and considering its widespread use and environmental persistence, further investigations are warranted to clarify the long-term metabolic consequences of CPF exposure, particularly in vulnerable populations chronically exposed through diet.

## Data Availability

The original contributions presented in the study are included in the article/**Supplementary Material**. Further inquiries can be directed to the corresponding author.
