# Supplementary material for: The endocrine disruptor chlorpyrifos alters hypothalamic Npy and Agrp expression via ERβ-dependent regulation in vitro and in vivo
Source: Front Endocrinol (Lausanne). 2026 Jan 14;16:1726498. doi: 10.3389/fendo.2025.1726498 (PMC12846974; doi:10.3389/fendo.2025.1726498)
Supplement: Supplementary file 1 [file DataSheet1.pdf]

Supplementary Material

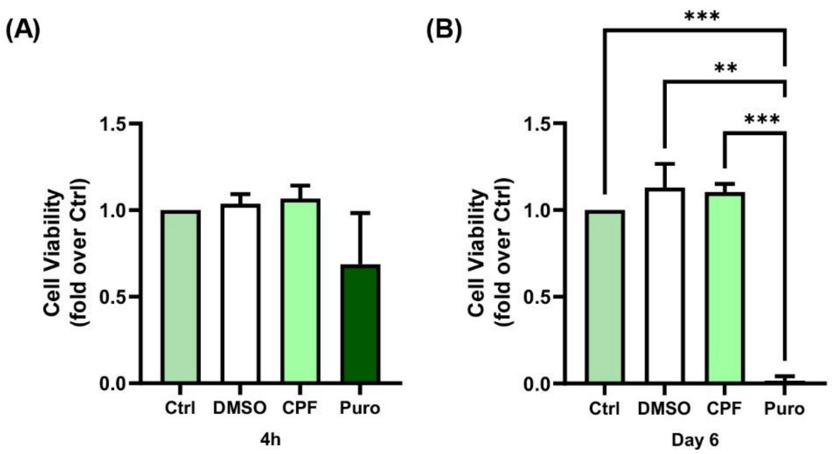

Supplementary Figure 1. Evaluation of cytotoxicity in CPF-treated mHypoE-N46 cells. Cell viability was measured by sulforhodamine B assay following acute (4 h) **(A)** and chronic (Day 6) **(B)** exposure to CPF (1 pM). DMSO was used as vehicle, and puromycin (2 µg/mL) was included as a positive control for cytotoxicity. Statistical significance was assessed using one-way ANOVA followed by Tukey multiple comparisons test (**$p < 0.01$, ***$p < 0.001$).

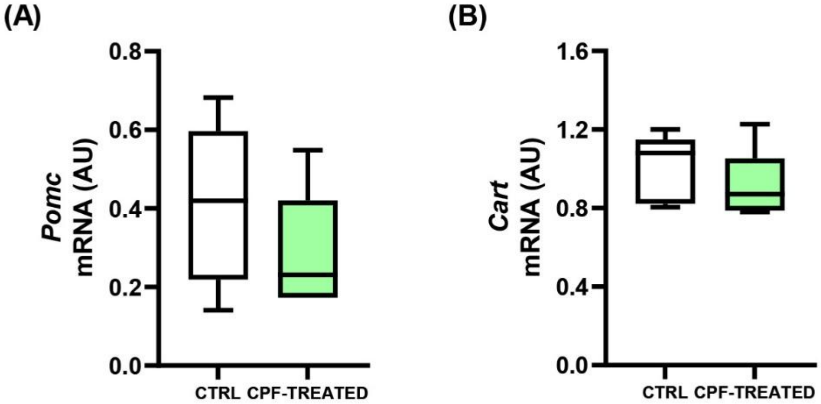

Supplementary Figure 2. Effects of CPF exposure on hypothalamic anorexigenic gene expression in mice. Hypothalami were collected from 6-month-old F2 male CD-1 mice chronically exposed to CPF (10 mg/kg/day) from conception to 6 months of age while maintained on a standard diet. **(A-B)** *Pomc* and *Cart* mRNA levels were quantified by qPCR. Data are expressed as absolute units (AU) and presented as boxplots showing mean values from n = 5 mice per group. Normality was assessed using the Shapiro–Wilk test. No statistically significant differences were detected between groups.
